# Cetuximab in small bowel adenocarcinoma: a new friend?

**DOI:** 10.1038/sj.bjc.6605898

**Published:** 2010-09-14

**Authors:** D Santini, M E Fratto, C Spoto, A Russo, S Galluzzo, A Zoccoli, B Vincenzi, G Tonini

**Affiliations:** 1 Medical Oncology, University Campus Bio-Medico, Via Alvaro del Portillo, 200, Rome 00128, Italy; 2 Department of Surgical and Oncological Sciences, University of Palermo, Via del Vespro 179, Palermo 90127, Italy

**Sir,**

Small bowel adenocarcinoma (SBA) is a rare and aggressive tumour. SBA in the United States increased from 5.7 cases per million in 1973 to 7.3 cases per million in 2004 (Surveillance Epidemiology and End Results (SEER), 1973–2004 database; Jemal *et al* (2009). Surgery is the mainstay of treatment, even if chemotherapy in advanced disease has been associated with an increased survival. The most effective agents include 5-FU, irinotecan, platinum agents and gemcitabine (Fishman *et al*, 2006; Speranza *et al*, 2010). The molecular characterisation of this cancer could help to improve prognosis. Specifically, the frequency of *KRAS* gene mutations is similar than in colorectal cancer (Ari *et al*, 1997). The role of targeted therapy, specifically of epidermal growth factor receptor (EGFR) inhibitors, has never been investigated in patients with SBA. A recent report showed that a high percentage of tumours express both EGFR and VEGF-A suggesting that these patients could benefit from therapeutic strategies targeting EGFR and VEGF receptors (Overman *et al*, 2010).

In our institution four patients with advanced SBA (three men and one woman) were enrolled to receive cetuximab in combination with chemotherapy. The primary site of disease was duodenum in two patients and jejunum in the other two patients. All patients had peritoneal carcinosis, whereas one presented also bone, abdominal lymph nodes and liver metastases. Cetuximab (250 mg mq^−1^, with a loading dose of 400 mg mq^−1^) was associated with CPT-11-based chemotherapy in first- (two patients) or second-line (two patients) therapy for metastatic disease. The patients previously treated progressed with folfiri during first-line chemotherapy. The median number of weekly cetuximab cycles was 14 (range: 8–28). One patient obtained a complete peritoneal response, two patients a partial peritoneal response and one patient stable disease in all the disease localisations. Two patients have a time to progression with cetuximab of 3 months, whereas the other two are still on cetuximab after 3 and 10 months. Two patients are still alive (overall survival of 7 and 17 months), the other two had an overall survival of 35 and 19 months. Treatment was well tolerated, with one patient presenting grade 3 neutropenia and grade 3 diarrhoea. Nobody needed to have a dose modification. In three patients *KRAS* status was tested, resulting in *KRAS* wild-type mutational status. The same patients presented a G2 skin rash, that was associated with the two partial responses and the complete response. Table 1 shows the main characteristics and the results.

These case reports show that anti-EGFR therapy may have a role in SBA, especially in those patients harbouring a wild-type KRAS status. A prospective trial is needed to explore and to support these preliminary observations.

## Figures and Tables

**Table 1:** Patients’ characteristics and results obtained with cetuximab

Total number	4
	
*Age (years)*	
Median (minimum–maximum)	64.25 (57–61)
	
*Gender, number* (%)	
Male	3 (75)
Female	1 (25)
	
*Primary site* (%)	
Duodenum	2 (50)
Jejunum	2 (50)
	
*Grading, number* (%)	
Well differentiated	0 (0)
Moderately differentiated	1 (25)
Poorly differentiated	2 (50)
	
*KRas status*	
Wild-type	3 (75%)
Mutant	0 (0%)
Not available	1 (25%)
	
*Response to cetuximab*	
Progression	0 (0%)
Stable disease (second line)	1 (25%)
Partial response (first and second line)	2 (50%)
Complete response (first line)	1 (25%)
	
*Skin rash*	
G0	1 (25%)
G1	0 (0%)
G2	3 (75%)
